# The Impact of COVID-19 On Comorbidities: A Review Of Recent Updates For Combating It

**DOI:** 10.1016/j.sjbs.2022.02.006

**Published:** 2022-02-10

**Authors:** Jonaid Ahmad Malik, Sakeel Ahmed, Mrunal Shinde, Mohammad Hajaj Said Almermesh, Saleh Alghamdi, Arshad Hussain, Sirajudheen Anwar

**Affiliations:** a Department of Pharmacology and Toxicology, National Institute of Pharmaceutical Education and Research, Guwahati, India; b Department of Biomedical Engineering, Indian Institute of Technology Ropar, Rupnagar, India; c Department of Pharmacology and Toxicology, National Institute of Pharmaceutical Education and Research, Ahmedabad, India; d Department of Pharmaceutical Analysis, National Institute of Pharmaceutical Education and Research, Guwahati, India; e Department of Pharmacology and Toxicology, College of Pharmacy, University of Hail, Hail, Saudi Arabia; f Department of Clinical Pharmacy, Faculty of Clinical Pharmacy, Al Baha University, Al Baha, Saudi Arabia; g Department of Clinical Pharmacy, College of Pharmacy, University of Hail, Hail, Saudi Arabia

**Keywords:** Comorbidities, SARS-CoV-2, Complications, Variants, Mortality, **AD**, Alzheimer’s Disease, **ACE-2**, Angiotensin-converting enzyme-2, **AMI**, Acute Myocardial Infarction, **AIA**, Avian Influenza A, **HCQ**, Hydroxychloroquine, **ANE**, Acute Necrotizing Encephalopathy, **ARBs**, Angiotensin receptors blockers, COPD, chronic obstructive pulmonary disease, **CAP**, Community-Acquired Pneumonia, **CVS**, Cardiovascular, **CVDs**, cardiovascular diseases, **CDCP**, Centres for Disease Control and Prevention, **CF**, Cardiac failure, **CHD**, Coronary heart disease, **DM**, Diabetes Mellitus, **DIC**, Disseminated intravascular coagulation, **FLAIR**, Fluid-Attenuated Inversion Recovery, **GBS**, Guillain-Barre Syndrome, **G+C**, Guanine + Cytosine, **HLH**, Hemophagocytic Lymphohistiocytosis, **HT**, Hypertension, **ICUs**, Intensive care units, **IVIG**, Intravenous immunoglobin, KIMII-Kawasaki-identical to multisystem inflammatory illness, KMS, Kasabach-Merritt Syndrome, **MI**, Myocardial infarction, NDs, Neurological disorders, **NSTEMI**, Non-ST-elevated myocardial infarction, **PCI**, Percutaneous Coronary Intervention, **PPE**, Personal protective equipment, **pRb**, Retinoblastoma protein, **RNS**, Reactive Nitrogen Species, BP, Blood pressure, ROS, Reactive oxygen species, SEM, Scanning **electronic** microscope, SARS-CoV-2, severe acute respiratory syndrome coronavirus 2, **SIRS**, Systemic inflammatory response syndrome, **STEMI**, ST-elevated myocardial infarction, **PD**, Parkinson’s Disease

## Abstract

Coronavirus disease is caused by the SARS-CoV-2 virus. The virus first appeared in Wuhan (China) in December 2019 and has spread globally. Till now, it affected 269 million people with 5.3 million deaths in 224 countries and territories. With the emergence of variants like Omicron, the COVID-19 cases grew exponentially, with thousands of deaths. The general symptoms of COVID-19 include fever, sore throat, cough, lung infections, and, in severe cases, acute respiratory distress syndrome, sepsis, and death. SARS-CoV-2 predominantly affects the lung, but it can also affect other organs such as the brain, heart, and gastrointestinal system. It is observed that 75 % of hospitalized COVID-19 patients have at least one COVID-19 associated comorbidity. The most common reported comorbidities are hypertension, NDs, diabetes, cancer, endothelial dysfunction, and CVDs. Moreover, older and pre-existing polypharmacy patients have worsened COVID-19 associated complications. SARS-CoV-2 also results in the hypercoagulability issues like gangrene, stroke, pulmonary embolism, and other associated complications. This review aims to provide the latest information on the impact of the COVID-19 on pre-existing comorbidities such as CVDs, NDs, COPD, and other complications. This review will help us to understand the current scenario of COVID-19 and comorbidities; thus, it will play an important role in the management and decision-making efforts to tackle such complications.

## Highlights

• SARS-CoV-2 can also affect organs other than the lungs, including the brain, heart, and gastrointestinal system.
• Patients with Cancer, HIV, COPD, neurological, and CVDs are more prone to the COVID-19 associated complications, leading to a drastic rise in morbidity and mortality.
• Elderly and pre-existing polypharmacy patients have worsened COVID-19 associated complications. When a person with comorbidity is infected with SARS-CoV-2, it becomes more dangerous, and managing these patients with adequate medical care is critical to their survival.
• A co-morbid person should adhere to preventive measures to reduce mortality, including regular handwashing with soap or using an alcohol-based hand sanitizer, minimizing in person contact and practicing social distance, wearing a face mask in public places, and avoiding going to public places unless essential are among the precautional measures.

## Graphical abstract

**Figure:**
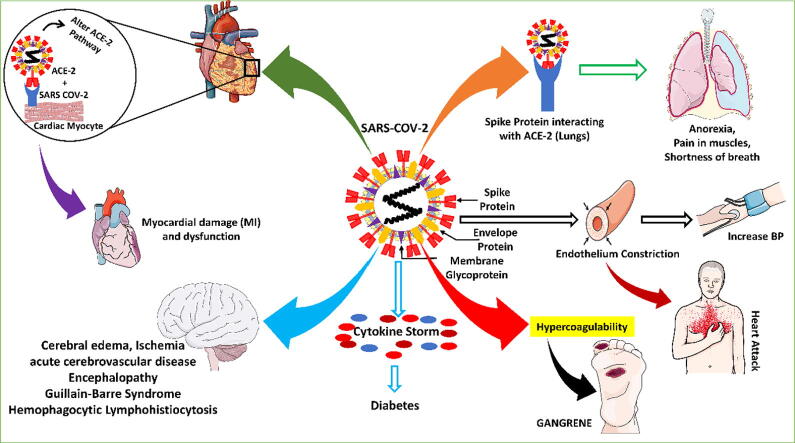

## 1 Introduction

Over the last few decades, the globe has witnessed the birth of novel viruses that have presented severe health risks worldwide (Malik and Maqbool, 2020). The SARS-CoV-2 virus, formerly known as a novel coronavirus, broke out in Wuhan (China) and caused major morbidity and mortality globally (Malik et al., 2021; Guan et al., 2020). It was first appeared in late 2019 and caused Corona Virus Disease-19 (COVID-19). The WHO declared the SARS-CoV-2 epidemic on 11th March 2020. It has affected 269 million peoples in 224 nations and territories, with 5.3 million deaths, mainly affecting elders and front-line workers. COVID-19 instances increased daily after the discovery of Omicron, reaching 0.6 million cases and thousands of deaths (Malik et al., 2022). Historically, in February 2003, the first SARS-CoV was found in China.

At present, seven types of coronavirus have been associated with human disease: OC43, NL63,229E, HKU1, SARS-CoV, MERS-CoV, and the most recent one is SARS-CoV-2 (Malik et al., 2022). The general symptoms of COVID-19 include fever, sore throat, cough, lung infections, and, in severe cases, acute respiratory distress syndrome, sepsis, and death. SARS-CoV-2 predominantly affects the lung, but it can also affect other organs such as the brain, heart, and gastrointestinal system. Fever is a major symptom in other infections, like incase of urinary tract infections (Alzahrani et al., 2021). It is observed that 75 % of hospitalized COVID-19 patients have at least one COVID-19 associated comorbidity. The most common reported comorbidities are hypertension, NDs, diabetes, cancer, COPD, endothelial dysfunction, and CVDs Table 2.

Moreover, older and pre-existing polypharmacy patients have worsened COVID-19 associated complications. SARS-CoV-2 also results in the hypercoagulability issues like gangrene, stroke, pulmonary embolism, and other associated complications (Sanyaolu et al., 2020). Diabetic individuals have higher morbidity and mortality and higher hospitalization and ICU admittance rates (Sanyaolu et al., 2020). The mortality rate is also higher in those with CVDs with elevated troponin levels (Böhm et al., 2020). On the other side, SARS-CoV-2 is more likely to cause severe illness in COPD (4-folds higher prevalance) or other respiratory disorders patients. The geriatric population, especially those with NDs, could manifest flulike symptoms. As a result, COVID-19 should be included in the differential diagnosis and management of individuals with neurologic problems, particularly in severely infectious areas (Mao et al., 2020; Albanghali, 2022). Moreover, cancer patients are more susceptible to SARS-CoV-2 contamination due to immunocompromised status caused by either malignancy or intervention of anti-neoplastic drugs, radiotherapy, hormonal therapy, and surgery (HJ et al., 2021).

Interestingly, pediatric cancer patients show relative resistance to SARS-CoV-2 infection. This further supports the fact that age is more important for SARS-CoV-2 infection than cancer (Derosa et al., 2020). Like oncoviruses, SARS-CoV-2 causes substantial carcinogenic inflammation (Geisslinger et al., 2020). However, there is a lack of evidence to conclude whether it possesses the tumorigenic ability or not. As the emergence of Omicron again puts all the scientific community in big trouble, it is of utmost importance to look at the possible risk of co-morbid patients with COVID-19. It is essential to understand how co-morbid conditions increase the chance of SARS-CoV-2 infection subsequently increase mortality among elderly patients. It is an emergent need to take precautionary measures to avoid morbidity and mortality. The present review demonstrates the impact of COVID-19 on comorbidities. Information provided in the review will play an important role in the management and decision-making efforts to tackle such complications to reduce the further burden of the COVID-19 pandemic in the older population with pre-existing comorbidities.

## 2 Impact of COVID-19 on the CVS complications

The major concern of cardiologists is to conclude whether people with CVDs are at a greater risk for SARS-CoV-2. Various studies have established the association between CVS disorders with MERS and SARS infection (Badawi and Ryoo, 2016). Analysis of 637 MERS-CoV revealed that 50 % of cases have a high prevalence of diabetes and high blood pressure, and 30% of cases have a high risk of cardiovascular ailments (Badawi and Ryoo, 2016; B. Li et al., 2020a).

The interaction of SARS-CoV-2 with ACE-2 receptors (largely expressed in the lungs, heart, GIT system, kidneys) is well documented. It is found that with the help of ACE-2 receptor interaction, virus reaches cardiac myocytes and epithelial cells lining the alveolar tissue (Soumya et al., 2021). Moreover, ACE-2 has a role in the neurohumoral regulation of the CVS. The engagement of SARS-CoV-2 with cardiac and alveolar ACE-2 resulted in alteration of ACE-2 signaling that leads to acute injury to the lungs and heart (Samavati and Uhal, 2020, Farooqi et al., 2021) ACE-2 shields the heart from the innervation of RAAS, which is involved in the conversion of Angiotensin-II (Ang II) to Angiotensin (I-VII). Ang II is a powerful vasoconstriction with proinflammatory activity that induces capillary endothelial damage, while Angiotensin (I–VII) has opposite action. The entry of the virus down-regulates the ACE-2 and elevates levels of Ang II, which enhanced the risk of cardiac injury. Therefore, elevated ACE-2 receptors will enhance the virus content but have a cardiprotective potential. There is an alarming escalation in comorbidities among CVD patients. The infection intervenes with biochemical pathways relevant to the CVS like ACE-2 pathway, cardiac muscle integrity, fibrinogen pathways, redox homeostasis, induces breakage of plaques present in the stent, and finally, aggravates myocardial damage and dysfunction (Groß et al., 2020).

**Fig. 1:**
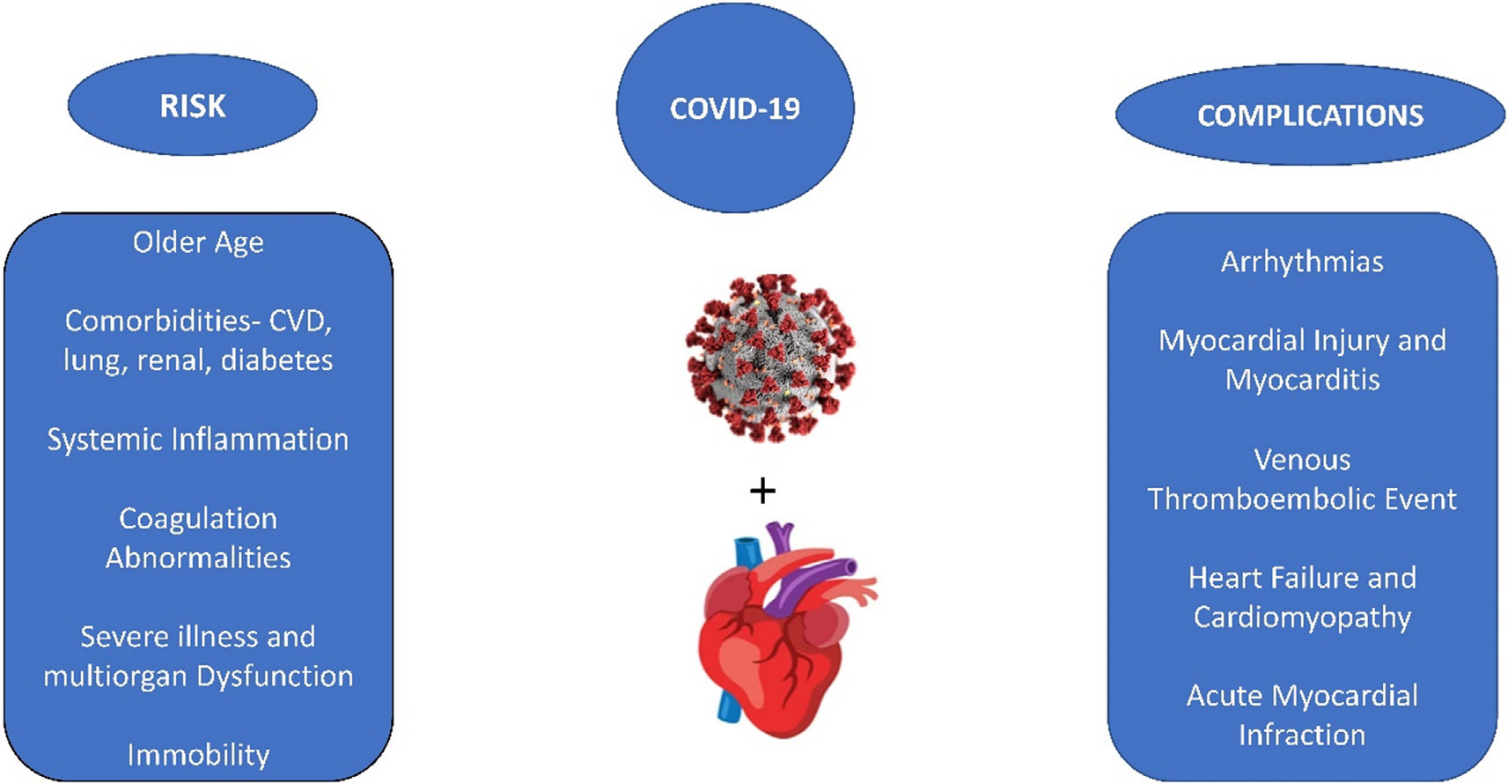
Risk and the complications of COVID-19 associated with cardiovascular disease.

Mild heart injury and persistent CVS damage are basic CV illnesses associated with SARS-CoV-2 infection. According to the reports, 7.2–19.7% of patients, including 40% of patients with CV and cerebrovascular illnesses, suffer from acute cardiac injury Table 1 (G et al., 2020). A retrospective study with 1009 COVID-19 patients showed that 14.9 % of the subjects suffered from HT, and 2.5 % suffered from CHD (Shi et al., 2020). Further more a study reported that 72,314 COVID-19 participants found a 10.5 % death rate among individuals with CVDs (Zhou et al., 2020). Patients with HT, CHD, DM, and HF are more prone to COVID-19 infection, especially in the geriatric population. The patients with the comorbidities mentioned above have a greater need for ICU admittance, and the mortality rate was also elevated among seriously affected individuals. Several reasons have contributed to this, including inflammatory response, discrepancies among rising O_2_ consumption and hypoxic conditions, and unbalanced plaque break caused by blood flow variations. Fig. 1 shows the risk and the associated complications of COVID-19 with cardiovascular diseases in patients with MI.

**Table 1:** Clinical data study for the impact of SARS-CoV-2 on comorbidities.

**Patients (No.)**		**Age (years)**	**Comorbidities %**				**References**
**All**	**Male**		**HT**	**DM**	**RD**	**CVD**	
41	30	49.0	15.0	20.0	2.0	15.0	(Yang et al., 2020)
137	61	57.0	9.5	10.2	1.5	7.3	(K. Liu et al., 2020)
12	8	53.7	25.0	16.7	8.3	33.3	(Y. Liu et al., 2020)
138	75	56.0	31.2	10.1	2.9	14.5	(Bai et al., 2020)
140	71	57.0	30.0	12.1	1.4	5.0	(J. jin Zhang et al., 2020)
9	5	35.2	0	11.1	0	0	(MQ et al., 2020)
1099	640	47.0	14.9	7.4	1.4	2.5	(Guan et al., 2020)

**Table 2:** SARS-CoV-2 and Comorbidities.

S. No.	Disease	SARS-CoV-2 targets/Mechanism	Symptoms/Syndrome	References
1	Hypertension	Overexpression of ACE-2 receptor	Blood pressure increased	(L et al., 2020).
2	Cardiovascular Diseases	Impaired immune system (patients experience inflammation in the cardiac muscles), Elevated troponin level, Interaction of the SARS-CoV-2 with ACE-2 in cardiac myocyte	Myocardial infarction, heart attack, dysrhythmia	(Sprockel et al., 2021)
3	Neurological Complications	Inflammatory response and hypercoagulation, enhanced D-dimers, prolongation of prothrombin time and DIC	Acute Cerebrovascular Disease Encephalopathy GBS HLH	(Tang and Hu, 2021, Uginet et al., 2021)
4	Liver diseases	ACE-2 and TMPRSS2 expression in liver cells	Elevated serum alanine aminotransferase (ALT) and aspartate aminotransferase (AST)	(Marjot et al., 2021)
5	Renal diseases	Imbalance of the Renin-Angiotensin System (RAS), Increased levels of dipeptidyl peptidase-4 and ACE-2	Acute kidney injury (AKI) (sudden loss of kidney function)	(Bitencourt et al., 2020)
6	Endothelial dysfunction	Immune-inflammatory responses, expression and function of its receptor angiotensin-converting enzyme 2 (ACE2) in the vasculature	Inflammation-induced heart failure	(Sisti et al., 2021)
7	HIV	Impaired immune response and ACE-2 receptor in the lungs	Jaundice A low CD4 count	(Ssentongo et al., 2021)
8	Obesity	The abnormal cytokines secretions and adipokines	Chronic obesity with effect on bronchi and lung parenchyma	(Simonnet et al., 2020)
9	Stroke	Hypercoagulability, endothelial injury, vasculitis	Shaking with chills	(Qureshi et al., 2021, Spence et al., 2020)
10	Diabetes	ACE-2 expression, cytokines storm	Pneumonia like symptoms Blood counts of IL-6, C.R.P., and ferritin	(Maddaloni and Buzzetti, 2020)
11	Gangrene	COVID-19 associated hypercoagulability	Localized death, decomposition, and putrefaction of toe or foot fingers	(E et al., 2020)
12	Pulmonary diseases Asthma	local/systemic inflammation, compromised host response, overexpression of ACE-2 receptor in lungs cells	Shortness of breath, cough, pneumonia (2.5-fold more risk), Severe hypoxemia	(Dong et al., 2020; Qiu et al., 2020)
13	Cancer	Immune dysregulation and chronic inflammation, increase in cytokine levels including IL-6	Adult respiratory distress syndrome	(Jyotsana and King, 2020; Wang et al., 2020b)

### 2.1 Impact of COVID-19 on adults with CVDs

Patients with CV comorbidities are more prone to SARS-CoV-2 infection (such as coronary artery disease and HT), and difficulties such as ARDS (Zhou et al., 2020). Individuals with CVDs had a greater death rate with SARS-CoV-2 infection. SARS-CoV-2 contamination can exacerbate MI and necrosis, aggravating myocardial infarction and ultimately HF (JP et al., 2020). The exact mechanism contributing to heart injury in COVID-19 individuals is unknown but assumed to be the involvement of ACE-2 (F et al., 2020). In a mouse model, lung infection produced ACE-2 dependent cardiac complications in subjects with SARS-CoV-2 infection (Sun, 2020). In Toronto, post mortem analysis of SARS-CoV-2 patients revealed SARS coronavirus RNA's existence in heart samples. Other studies demonstrated that SARS-CoV-2 associated cardiac complications are distinguished by a cytokine crisis caused by disbalance in helper T-cell subtype responses and intracellular calcium overload due to hypoxia, which leads to cardiomyocyte death. Underlying CV diseases may be more mutual in the geriatric subjects, persons with weakened immunological systems, high ACE-2 concentrations (Ejaz et al., 2020).

### 2.2 Impact of COVID-19–2 on adolescents with CVDs

Adolescents with COVID-19 seem to have lenient symptoms of COVID-19 than adults. Manifestations of irregular HR and pneumothorax have been documented in neonates born to perinatal COVID-19 infected moms. Adolescents predominantly seem more susceptible to other coronaviruses, which could make them immune to SARS-CoV-2 infectivity, at least in part. The mechanisms and causes are not well understood. It is believed that one of the mechanisms might be differential ACE-2 functions in adults and children, which is stronger in adults than children; SARS-CoV-2 is highly contagious in grown-ups (Li et al., 2021). Moreover, maternal antibodies have been shown to protect newborns and infants against bacterial and viral infections (VDL et al., 2017). Immune systems of newborns and young children have memory B, and T cell pools, preventing reinfection from common pathogen. As a result, adolescents’ immune systems are often more capable of responding to novel germs than adults. However, these functions may be diminished with the advancement of age and in patients over 70 years old, they may even be ineffective (C et al., 2020). Throughout the COVID-19 pandemic in Paris, a team led by Martin Chalumeau at the University of Paris examined adolescents' medical aspects with KIMII. SARS-CoV-2 may be linked to the ongoing epidemic of KMSI disease in adolescents in Paris (Toubiana et al., 2020). The majority of adolescents, especially those of African heritage, develop gastrointestinal indications and Kawasaki disease shock syndrome (Chen et al., 2020a).

### 2.3 Impact of COVID-19 on myocarditis

A study with 41 COVID-19 subjects in Wuhan concluded that 12 % of subjects had an elevated troponin level (cut-off of 28 pg/mL), representing one of the earliest cardiac damage linked to SARS-CoV-2. In later trials, cardiac injury associated with an elevated troponin concentration was seen in several hospitalized COVID-19 subjects, and 22–31% were admitted to the ICU (Sprockel et al., 2021). Myocarditis also has been linked to elevated virus load with mononuclear invasion in the autopsy samples of COVID-19 patients (Long et al., 2020), which accounts for 7% of COVID-19-related fatalities (Ruan et al., 2020).

### 2.4 Impact of COVID-19 on AMI and chronic MI

In COVID-19 patients, cardiac injury is manifested in multiple ways. Contamination, inflammation and febrile conditions turn the vascular system more vulnerable to clot formation and interferes with the body's ability to dissolve a clot. Despite arteries being devoid of fatty acids calcified flow-limiting blockages, chances of cardiac injury similar to the injury induced by a heart attack (MI type 2) (Böhm et al., 2020). The pathology occurs when there is a lack of oxygen supply to the cardiac myocytes, one of the predominant clinical conditions associated with SARS-CoV-2 infection (J et al., 2020). During fever and inflammation, the oxygen demands of various organs get increased. Suppose the infection is localized in the lung, the stress level increases, affecting the gaseous exchange, resulting in a drastic reduction in the supply of O_2_ to the cardiac muscles. Since the virus targets the heart, COVID-19 positive patients experience inflammation in the cardiac muscles, along with those individuals who had been formerly healthy with no heart problems. This very characteristic of the inflammatory pathway leads to damage to the cardiac muscle, dysrhythmia, and heart failure (Soumya et al., 2021). High systemically mediated inflammation increases both atherosclerotic plaques breakdown and AMI. In a study, viral infections were associated with an elevated risk of AMI between the first seven days of diagnosis of the illness, with 6.1 being the incidence ratio for influenza and other viruses having a 2.8 ratio (Kwong et al., 2018). COVID-19 individuals are at higher risk for AMI due to significant inflammatory responses and hypercoagulability (Qureshi et al., 2021). The therapy of AMI in COVID-19 subjects remains questionable. While fibrinolysis may be contemplated in individuals with a STEMI with COVID-19. According to the ACC, fibrinolysis should be avoided in those with “low-risk STEMI.” Several facilities conduct PCI more frequently, and it remains the therapy of preference for lower STEMI with no right ventricular inclusion or lateral AMI, mostly with no hemodynamic instability. If PCI is done, personnel must adopt suitable PPE, and the catheterization laboratories must be fully disinfected. Individuals with NSTEMI who are hemodynamically vulnerable must be addressed in the same way STEMI individuals (Jain et al., 2021).

### 2.5 Impact of COVID-19 on cardiomyopathy

In acute Congestive failure (CF), COVID-19 contamination is the predominantly manifested. Acute HF evidents in 23 % of COVID-19 subjects at the time of diagnosis, with cardiomyopathy appearing in 33% of the individual (Wu and McGoogan, 2020). A study reported that HF was detected in 24 % of subjects and associated with an increased fatality rate. Almost half of the patients with HF had no prior history of HTN or CVD (C et al., 2020). It is unknown whether this HF results from nascent cardiomyopathy or deterioration of formerly undiscovered HF (Böhm et al., 2020). Right HF can also happen, especially in a population with acute respiratory distress syndrome and acute lung injury (L et al., 2020).

### 2.6 *Impact of COVID-19 on hypertension*

It is unclear whether elevated BP in an uncontrolled manner is a risk factor for acquiring COVID-19 or whether controlled blood pressure among patients with hypertension. Lippy et al. pooled analysis demonstrated a 2.5-fold more risk of lethality in COVID-19 with high BP, predominantly in geriatric patients. During the infection, the ACE-2 receptor mediates the entry of the virus into the lung, and patients with high blood pressure have more devastating results than other clinical conditions (Lippi et al., 2020). Drugs like ACE-2 inhibitors and ARBs were administered to patients with CVS disorders, including congestive heart failure and hypertension. The administered drugs lead to overexpression of ACE-2, thus resulting in an increased risk of devastating COVID-19. Fang et al. (2020) proposed that subjects administered with ACE-2 elevating drugs for hypertension, diabetes, or cardiac diseases have a higher risk of SARS-CoV-2 infection and, therefore, should be monitored for these medications (L et al., 2020). On the contrary, data collected from several cardiological societies support the administration of ACE inhibitors or ARBs in chronically elevated blood pressure in COVID-19 hospitalized patients (P. Zhang et al., 2020).

## 3 Impact of COVID-19 on neurological diseases

Although SARS-CoV-2 is considered a respiratory pathogen, many neurologic complications, including confusion, stroke, and neuromuscular disorders, also manifest during acute COVID-19. Furthermore, disorders such as impaired concentration, headache, sensory disturbances, depression, and even psychosis may persist for months after SARS-CoV-2 infection, as part of a constellation of symptoms now called Long COVID. Even young people with mild disease can develop acute COVID-19 and Long COVID neuropsychiatric syndrome (Spudich and Nath, 2022). The structural features of the human corona virus and the mechanism of inducing infection make it a potential host for CNS (Chen et al., 2020b; Y. C. Li et al., 2020). The exact mechanism of the human corona virus entering the CNS remains unclear. The distribution of ACE-2 receptors in the neuronal tissue is insufficient to describe viral neurotropism (Y. C. Li et al., 2020). Another possible mechanism could be axonal transport, which leads to neuronal damage (Dubé et al., 2018). The aerosol droplets facilitate the human corona virus to enter the nasal mucosa of the infected host; thereby, the virus gains access to the CNS (Y. C. Li et al., 2020). Once in the CNS, the membrane-bound ACE-2 receptor, ubiquitously present in cerebral capillary endothelium, glial cells, and neurons, assures SARS-CoVs to fuse with cell surface via spike proteins (Baig et al., 2020). Strong adhesion subsequently leads to further axonal transport resulting in the spread of infection to the piriform cortex and other regions associated with olfaction (Dubé et al., 2018). Within days after the viral entry, it diffuses into the CNS and is observed in the neuronal region of infected mice or healthy subjects after the acute manifestation of the infection (Y. C. Li et al., 2020).

### 3.1 Pathophysiology and clinical manifestations

SARS-CoV-2 comes under the family of beta-coronaviridae. The identifying features of this virus include an envelope, non-segmented, single-stranded, positive-sense RNA. (Jin et al., 2020). The multifaceted pathways through which the virus inflicts neurological damage directly injure particular receptors, like ACE-2, cytokine storm, 2° hypoxic injury, and anti-retrograde traveling to the nerve fibers (Hassett et al., 2020). Unlike lung epithelia, ACE-2 receptors are also expressed on the BBB endothelium that links the viral access to the CNS and damages the vascular system (Reynolds and Mahajan, 2021). The coalition of the SARS-CoV-2 with the lungs epithelia produces a universal SIRS that enhances the levels of IL-2, IL-6, IL-15, TNF- α; activation of glial cells leads to extensive production of proinflammatory state in CNS (Wu et al., 2020). Specifically, IL-6 levels correlate with the enhanced intensity of the COVID-19 illness (Wu et al., 2020). The systemic implications and alveolar injury cause severe hypoxia, leading to vasodilation in cerebral vessels, resulting in decompensated cerebral edema and ischemia (Guo et al., 2020). Eventually, the viruses proceed backward through the bulb and olfactory nerves, generating a pathway joining the epithelial cells in the nasal cavity and CNS which may also elucidate the common symptom of anosmia (Desforges et al., 2019).

Neurological manifestations in COVID-19 positive individuals have become more evident with the prior existence of neurological problems associated with more intense SARS-CoV-2 infections (Hassett et al., 2020). In a study, COVID-19 hospitalized patients, 8% had pre-existing neurological disorders, especially pre-existing strokes (Herman et al., 2020). Notably, within the first ten days of hospitalization, the extent of elevation of respiratory symptoms was lower in patients with pre-existing neurological complications. Moreover, there was a considerable increase in ARDS risk devoid of neurological complications (Mo et al., 2021). In another study where 179 subjects were diagnosed with SARS-CoV-2 pneumonia, prior cardiovascular complications significantly enhanced mortality (Ruan et al., 2020). An identical mortality trend was also observed in patients with PD (Silvia, 2020). A systemic review and *meta*-analysis indicated that there are 2.5 times higher risk of severe infection among subjects with pre-existing stroke (Nannoni et al., 2021).

Within the hospitalized group of patients, 6–36% of subjects had neurological manifestations (Hassett et al., 2020). Moreover, 20% of patients were inflicted with hypoxic-ischemic encephalopathy (Chen et al., 2020c). Rigorous efforts made to investigate neurotropism of the corona virus to address extensive brainstem-mediated manifestations in both pulmonary and cardiovascular systems (Nannoni et al., 2021).

### 3.2 Impact of COVID-19 on acute cerebrovascular disease with neurological indications

Although the etiology of COVID-19 is multifaceted, one of the most prevalent and significant neurological manifestations observed in COVID-19 patients includes acute cerebrovascular disease (Aggarwal et al., 2020). SARS-CoV-2 produce a universal inflammatory response and hyper coagulation, resulting from enhanced D-dimers, prolonging prothrombin time and DIC (Wang et al., 2020a).

In an Italian cohort, the rate of ischemic stroke was 2.5% in hospitalized COVID-19 patients, despite prophylaxis thromboembolism admission (Lodigiani et al., 2020). In comparison, the Chinese cohort reported a 5% higher rate of ischemic stroke. Comparably, in Dutch, the prevalence of ischemic stroke was found to be 3.7% in ICUs admitted patients despite the prophylaxis of thromboembolism (Ntaios, 2020). Notably, in younger patients, ischemic stroke with large vessel occlusions was reported (Oxley et al., 2020). Moreover, COVID-19 patients are prone to severe hypoxia in the cerebral region and infarcts, especially in patients with a prior cerebrovascular disorder (Wu et al., 2020). Inflammation and hyper coagulation can significantly enhance the chances of ischemic stroke, the greater risk associated with older patients (Siniscalchi et al., 2015). The protection of front-line workers during the evaluation of COVID-19 patients with stroke-like symptoms is of utmost importance. Various guidelines were proposed by the American Heart Association for a protected code of stroke, with special emphasis on screening guidelines, PPE, and crisis resource management (Khosravani et al., 2020). However, continuous medical care is required for patients diagnosed with ischemic stroke based on their institution laying special attention to intravenous thrombolytic medicaments and endovascular thrombectomy in the appropriate clinical scenarios without altering intervention criteria (Herman et al., 2020).

### 3.3 Impact of COVID-19 on PD and associated symptoms

Many people with PD are concerned about COVID-19 risk. Early reports describe worsening of parkinsonian symptoms during infection and poor prognosis (Schirinzi et al., 2020). Many PD-related symptoms worsened with COVID-19 infection. 18% of COVID-19 patients reported new motor symptoms, while 55% indicated worsening at least one previous motor symptom.) Non-motor symptoms were reported as new or deteriorating in all domains: mood (20 % new, 51 % worsening), cognitive (7.8% new, 41 % worsening), sleep (12 % new, 59 % worsening), and autonomic dysfunction (12 % new, 59 % worsening) (Brown et al., 2020). SARS-CoV-2 infection increased both motor and non-motor symptoms of PD, including stiffness, tremor, trouble walking, mood problems, cognition, and exhaustion. Viral infected PD patients report worsening PD symptoms, contribute to systemic inflammation, altered dopaminergic signaling, or changes in drug pharmacokinetics (Brugger et al., 2015). Direct infection of the CNS by SARS-CoV-2 is unlikely to worsen symptoms. Although COVID-19 has been linked to alterations in neuroimaging and SARS-CoV-2 RNA has been found in the cerebral fluid (Helms et al., 2020). Exacerbation of PD symptoms during COVID-19 could be partially attributed to the disease's inflammatory response (Chen et al., 2020b). The widespread occurrence of COVID-19-related symptoms exacerbation in PD patients underscores the need to consider COVID-19 as a possible explanation for rapidly increasing PD-related symptoms.

A higher percentage of women with PD affceted by COVID-19 than men. Women were not overrepresented in other case studies of patients with PD and COVID-19 (Cilia et al., 2020). COVID-19 has been shown to cause more severe disease in men than in women (Gebhard et al.,2020), but women may be more vulnerable.

### 3.4 Impact of COVID-19 on AD

COVID-19 has been shown to impact cognitive functions and potentially invade the brain, resulting in cognitive dysfunction (Xia et al., 2021). SARS-CoV-2, either directly or indirectly, impact AD patients. AD patients are more vulnerable to SARS-COV-2 infection as well as their their transmission (Mok et al., 2020). People in congregate living situations live in close quarters and share common areas (such as dining and living rooms), placing them at risk of infection. In addition, because older infected people may exhibit non-specific symptoms such as altered general activity, falls, or delirium rather than the typical COVID-19 symptoms of fever, cough, and difficulty breathing, their informal or professional caregivers may become infected if they do not take the necessary precautions (Nguyen et al., 2020, Rahman et al., 2021). AD patients may overlook simple infection-prevention measures such as hand washing, maintaining a social distance, and mask-wearing. The CDC has recommended strict risk to people with a history of dementia. They should take all precautionary measures, including reminders for regular hygiene practices, such as placing alarms in bathrooms to remind them to wash their hands with soap for 20 sec and wear a face mask (Iodice et al., 2021). AD patients have a higher frequency of emergency room visits, hospitalization, and mortality by SARS-CoV-2 infection than healthy aged adults (Hardan et al., 2021).

Long lockdowns and confinement during the pandemic plays an important role to impact neuropsychiatric problems in AD patients, particularly those with very low cognitive function. Social distancing exacerbated loneliness and affects mental health. The physical precautions implemented during the lockdown were more noticeable in patients with AD. These social and physical limitations impacted their mental health, and individuals with AD resulted in more neuropsychiatric disorders. Anxiety, depression, and hallucinations are major associated problems with AD patients. The length of confinement was found to have a positive link with the worsening of AD symptoms, with the longer the confinement, the greater the level of discomfort (Boutoleau-Bretonnière et al., 2020). Therefore, persons with severe cognitive impairments caused by AD are most vulnerable to unfavorable consequences during the lockdown. The loss of cognitive abilities and its impact on quality of life have major consequences for the family and caregivers. AD patients' caregivers also have a risk of infection and mortality (Chiao et al., 2015). Caregivers also take all precautionary measures to prevent the risk of infection and associated death.

### 3.5 Impact of COVID−19 on encephalitis and encephalopathy

Encephalitis is characterized by nausea, the onset of febrile conditions, convulsions, and unconsciousness (Jones, 1998). SARS-CoV-2 related encephalitis have been rarely found (Poyiadji et al., 2020). The pathophysiology remains unknown but is believed from secondary edema to inflammation-induced injury versus direct viral infection (Ye et al., 2020). ANE is a rare brain condition resulting from cytokine crisis and BBB damage, characterized by the absence of demyelination (Poyiadji et al., 2020; Mehta et al., 2020). Initially, a Non-contrast head CT scan illustrates symmetric, widespread lesions, whereas MRI with T2-weighted FLAIR shows hyperintense signal and internal hemorrhage (Breit et al., 2021). The most commonly affected regions are the thalamus, brainstem, cerebellum, cerebral white matter. ANE is more associated with influenza or zika infection, but this condition is also observed with SARS-CoV-2 (Wu et al., 2020).

### 3.6 Impact of COVID-19 on GBS

**Fig. 2:**
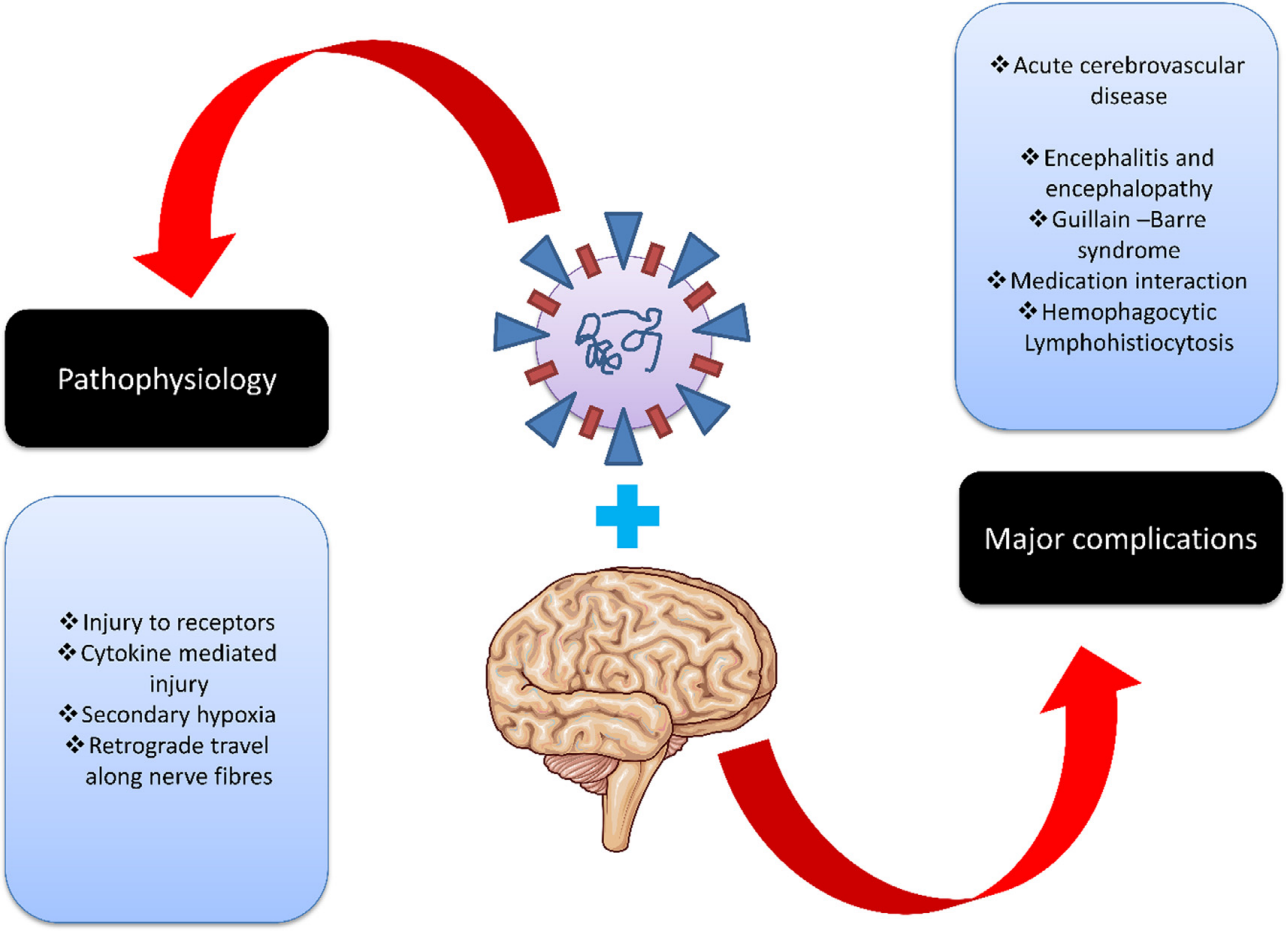
Major neurological complications associated with COVID-19.

GBS is a symmetrical, escalating flaccid paralysis, often caused by bacterial or viral illnesses of the pulmonary or GIT (Sejvar et al., 2011). This progressive neuropathy has been identified to be analogous with SARS-CoV-2 contamination, with five incidents found in Italy and two more incidents from Wuhan (China). All subjects felt a prelude of upper respiratory infections spanning from 1 to 14 days before the progression of symptomatic weakness; respiratory failure was reported in three patients. All subjects had a positive nose swab PCR test and lung scanning feature of SARS-CoV-2, but all CSF specimens were negative for SARS-CoV-2 (Toscano et al., 2020). Since all subjects were administered with IVIG, others that suffered pulmonary insufficiency fared poorly. Notably, brain and spine MRI failed to reveal discrepancies in 50 % of the patients, indicating the requirement of more profound tests and consultations, like studies based on the conduction of nerves, when there is a significant therapeutic concern even in the lack of radiological data (Sedaghat, 2020). Fig. 2 demonstrates the major neurological complications associated with COVID-19.

## 4 Impact of COVID-19 on diabetes Mellitus

Several pathophysiological explanations are linked DM with COVID-19. DM patients have an impaired innate immune organization, defense against SARS-CoV-2 (Guo et al., 2020). Furthermore, DM is a proinflammatory condition defined by an incorrect and excessive cytokine reaction, as demonstrated in COVID-19 subjects, where blood counts of IL-6, CRP, and ferritin were considerably greater in DM patients. This shows that persons with diabetes are vulnerable to an ICS, leading to shock, ARDS, and prompt COVID-19 infection. Furthermore, COVID-19 individuals with diabetes had greater D-dimer concentrations (Wang et al., 2020b). The hypercoagulation cascade in COVID-19 results in catastrophic thromboembolism and probable fatality in the context of a pre-existing latent pro-thrombotic hypercoagulable state predisposed condition exacerbated by the presence of DM (Wijaya et al., 2020). DM is linked to lower levels of ACE-2, subsequently decreased AT-II and to a lesser extent AT-I, especially AT I-7 and AT 1–9 individually. The respiratory ACE-2/AT 1-7 system has been demonstrated to possess anti-inflammatory and antioxidant characteristics, and ACE-2 has also been demonstrated to protect against deadly AIA H5N1 infections (Zou, 2014). As a result, the surge in prevalence of serious injury to lungs and ARDS associated with COVID-19 could be explained by reduced ACE-2 expression in DM. ARBs/ACEi are routinely utilized as anti-hypertensive and renoprotective medicines in persons with diabetes. Enhanced production of ACE-2 is linked to the utilization of ARBs/ACEi as an adaptable reaction to the increasing concentrations of AT-II (E et al., 2020). However, SARS-CoV-2 requires ACE-2 as a receptor as an entrance into the pneumocytes of the host cell. Therefore ACE-2 overexpression would make it easier for the coronavirus to enter and multiply. When the viruses use the enzyme to access the host tissue, ACE-2 is downregulated, and it can no longer defend the lungs from infection (Pal, 2020). According to a recent study, SARS-CoV-2 non-structural proteins target hemoglobin's b1-chain, causing iron to dissociate from porphyrin and decreasing hemoglobin's ability to deliver oxygen.

### 4.1 Impact of SARS-CoV-2 on pathophysiology of diabetes Mellitus

In Type 2 DM and Type 1 DM (T1DM) (particularly individuals who remain overweight and develop insulin resistance), COVID-19 can increase insulin resistance. Even modest COVID-19 can induce proinflammatory effects, seen by elevated IL-1b, IL-6, TNFα, MCP-1 & IP-10, leading to insulin resistance. Furthermore, overweightness, which is usually related to T2DM, increases the cytokine reaction, exacerbating resistance to insulin (Pal, 2020).

**Fig. 3:**
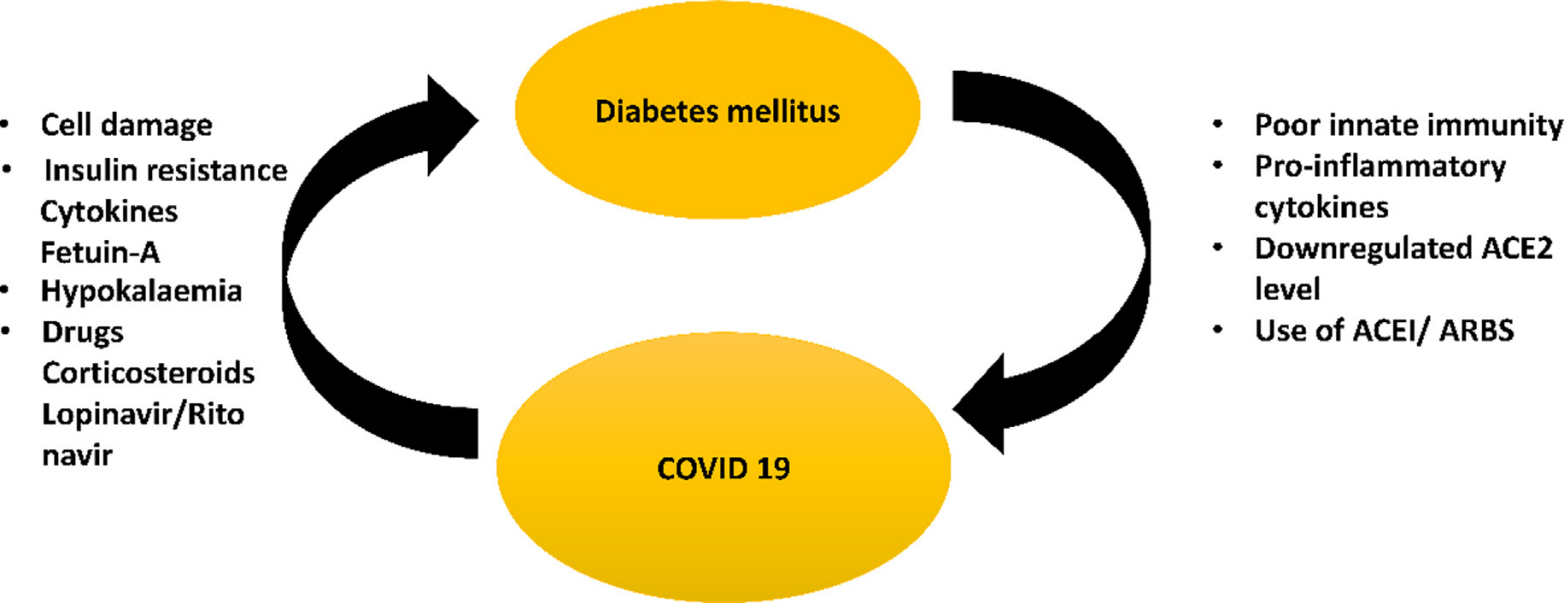
Representation of the mutual contact amongst the novel COVID-19 and DM. DM increases the seriousness of COVID-19 disease by compromising innate immunity, causing an excessive proinflammatory Cytokine reaction, and lowering ACE-2 expression. Besides, the usage ACEi/ARBs in patients with DM has been extensively linked to the intensity of disease severity in COVID-19. COVID-19, on the other hand, worsens sugar levels in persons with DM, possibly due to direct β-cell destruction mediated by viruses, increased resistance to insulin via fetuin-A and cytokines, and hypokalaemia. Furthermore, medications used to treat COVID-19, such as corticosteroids and lopinavir/ritonavir, might cause dysglycemia.

SARS-CoV-2 also raises serum concentrations of fetuin-A, an α2- Hermans-Schmid glycoprotein linked to insulin resistance (Yamasandhi, 2021). Finally, COVID-19 is frequently linked to hypokalaemia, decreased pulmonary ACE-2, angiotensin-II deprivation, and increased aldosterone secretion (Pal, 2020). Hypokalaemia, in turn, can exacerbate glucose regulators in T1DM and T2DM patients (Jones, 1998). Restriction on outdoor movements, along with the current state-wide lockdowns, would restrict sunlight exposure, resulting in vitamin D insufficiency. Insulin resistance has long been associated with hypovitaminosis D, which increases insulin sensitivity. As a result, vitamin D deficiency can impair glucose profiles in individuals later infected with COVID-19. It is also important to estimate the indirect effect of COVID-19 medicines on glycaemic control deterioration. Corticosteroids, commonly given in subjects with ARDS and infection, can cause hyperglycemia excursions. However, brief exposure in the current clinical context might not be clinically meaningful; lopinavir-ritonavir may cause lipodystrophy and consequent insulin resistance. More importantly, as ritonavir is an enzyme inhibitor, it can lengthen the t_1/2_ of glucocorticoids, indirectly contributing to an abnormal glycaemic profile (Epperla, 2015). Interferon-b1 (type 1 interferon) has also been found as a probable therapeutic approach for COVID-19, and interferon treatment has been linked to β-cell destruction. In COVID-19, Azithromycin was also utilized in conjunction with HCQ. The macrolide antibiotic can raise the likelihood of dysglycemia in DM patients (Pal, 2020). Data from Wuhan demonstrated that roughly 10 % of individuals with COVID-19 and T2DM experienced at least one incident of hypoglycaemic (3.9 mmol/L) episode in addition to worsening hyperglycemia (J.J et al., 2020). On the other hand, hypoglycemia provides an elevated incidence of (CV) episodes in the diabetic population by over-activating the SNS, mobilizing mononuclear cells that are proinflammatory, and raising platelet activity (A et al., 2019). Thus, COVID-19 worsens the glycaemic profile in patients with underlying DM, which further weakens the innate immune reaction and stimulates the production of proinflammatory cytokines, creating a chain of circumstances in following Fig. 3.

## 5 Impact of COVID-19 on cancer

A tumor is defined as any abnormal cells proliferation, which may be benign or malignant, and to make a distinction between the two is an important issue. A common skin wart, which remains confined to its location of origin, neither spread to distant organs nor attacks surrounding normal tissues, is considered a benign tumor. Unless benign tumor, the malignant tumor can invade surrounding and spread to distant body parts through the blood circulation and lymphatic systems, a property known as metastasis. In this context, only malignant tumors are referred to as cancers properly and because of their property of metastasizing, which makes this life-threatening and dangerous (Cooper and Hausman, 2007). The treatment received by cancer patients depends on the type of cancer a patient is exposed to and the advancement. Different approaches for treating different cancers include radiotherapy, surgery, chemotherapy, targeted therapy, immunotherapy, stem cell transplant, etc., but any treatment which shows therapeutic effect shows its adverse effects frequently (Zhang et al., 2015). The external therapies approach mainly depends on suppressing the immune system, which leads to risk of other opportunistic infections. Many studies have reported that most of the COVID-19 patients died from the disease had underlying conditions, and contagion is more deadly to those with underlying conditions than people with no diseases. CDCP explicitly states, ‘‘people of all ages with definite ‘comorbidities’ (cancers, COPD, obesity, CKD, HF and CHD (cardiac disorders), type 2 diabetes and sickle cell disease) are at higher risk of serious COVID-19 sickness”. A case study done in Wuhan revealed that people with cancer displayed more mortality rates due to SARS-CoV-2 contamination and the development of serious complications in a severe state of infection (L. Zhang et al., 2020). The Spanish Hospital reinforced this Chinese report that people with malignancies are more susceptible to COVID-19 with increment in serious complicacies. Treatment with a combination of Hydroxychloroquine and Azithromycin was suggested as an excellent therapy (R. J et al., 2020)

In India, a case study was done between 8th June to 20th August 2020 and reported that COVID-19 is elevated in cancer subjects, especially multiple underlying conditions, diabetes, and serious manifestations at presentations are highly correlated with COVID-19 related mortalities in the cohorts (A et al., 2021). Studies from the UK based on Coronavirus Cancer Monitoring Project published an article with interpretation concluding that fatalities from patients suffering COVID-19 in cancer subjects are gender-specific, underlying conditions, and age. Researchers were unable to find any indication that proves the people with malignancies on cytotoxic chemotherapy or any other anticancer therapy are at higher risk of mortalities due to COVID-19 illness (Lee et al., 2020). Moreover, COVID-19 induced inflammation could be reduced by chemotherapy given to cancer patients (R. J et al., 2020). With the rising cases of COVID-19 in India, new drug therapies (specifical corticosteroids) for combating the symptoms of disease cause other new iatrogenic diseases, e.g., Mucormycosis (black fungus), which is a rare opportunistic and fatal fungal infection (if incompletely treated). In India, cases of Mucormycosis have grown more gene expression and cell growth. Mucormycosis has been related more often with excessive use of corticosteroids in patients with diabetes and COVID-19 disease. A case study in India showed that uncontrolled corticosteroids in COVID-19 patients with diabetes increase the probability of catching fungal infections (Singh et al., 2021).

## 6 Impact of COVID-19 on HLH

HLH is a disease characterized by serious dysregulation of the NK cells, T-cells, and over-activation of macrophages, which eventually leads to cytokine storm with multiple organ failure (Al-Samkari and Berliner, 2018). However, this condition is associated with malignancy in the hematological cells, immune suppression, or severe contamination and has also been reported in COVID-19 patients. Clinical manifestations associated with HLH include dysfunction of the liver, elevated levels of triglycerides and ferritin, pancytopenia, remitting fever, and coagulopathy (Herman et al., 2020). Among the COVID-19 patients, HLH has become an unrecognized complication of an innate immune response by uncontrollable cytokine storm and enhanced quantity of IL-2, IL-6, IL-7, and TNF-α. Around 33% of COVID-19 patients suffered from HLH. Prompt diagnosis and scoring with HScore facilitate consideration of immunosuppressive therapies. Steroidal therapy and tocilizumab are investigated as a remedial choice for COVID-19 (Herman et al., 2020).

## 7 Impact of COVID-19 on gangrene

Gangrene is defined as the localized death, decomposition, and putrefaction of body tissues due to serious microbial infection or lack of blood supply to the organs. Gangrene is usually associated with the body extremities such as feet, toes, hands, or fingers but can affect any body part. Very few reports have been published which relates dry and intestinal gangrene with COVID-19, suggesting people developing dry gangrene in toes and fingers due to COVID-19 associated blood coagulation issues.

Earlier in this pandemic, Between 1st January and 15th March 2020, few dermatologists in Wuhan (China) and Lecco (Italy) respectively investigated the clinical and epidemiologic roles of COVID-19 in adult patients and reported no dry gangrene in serious COVID-19 subjects (De Giorgi et al., 2020). Other reports revealed that the geriatric population with comorbidities are more severely affected by COVID-19. COVID-19 patients on therapy for anticoagulation, the subject’s AVT progressed, leading to ischemic necrosis and dry gangrene of the lower extremities. Nonetheless, more research is needed to determine if coronavirus could induce circulatory system injury, thrombus, or necrosis (Liu et al., 2021).

The coronavirus has continuously evaluated a newer variant, a leading cause responsible for India’s worst COVID-19 s wave. Scientists suggested an intense pandemic has hit India, and it tends to produce a spectrum of rare symptoms, including gangrene or hearing loss in severe cases (“Doctors in India claim Covid variant is causing gangrene and deafness,” 2021). Clots, especially intestinal clots, have been experienced by COVID-19 patients, and physicians and surgeons have treated nearly a dozen patients. A patient vaccinated with both doses of Covishield was received by Andheri’s holy family hospital and, after scanning, showed multiple clots in the artery, which led to intestinal gangrene. The surgeon preferred anticoagulant therapy avoiding surgery, and it was the 10th such case in which COVID-19 and intestinal clot have presented together (“Covid Clots Now Causing Intestinal Gangrene - The Indian Practitioner,” 2021).

**Fig. 4:**
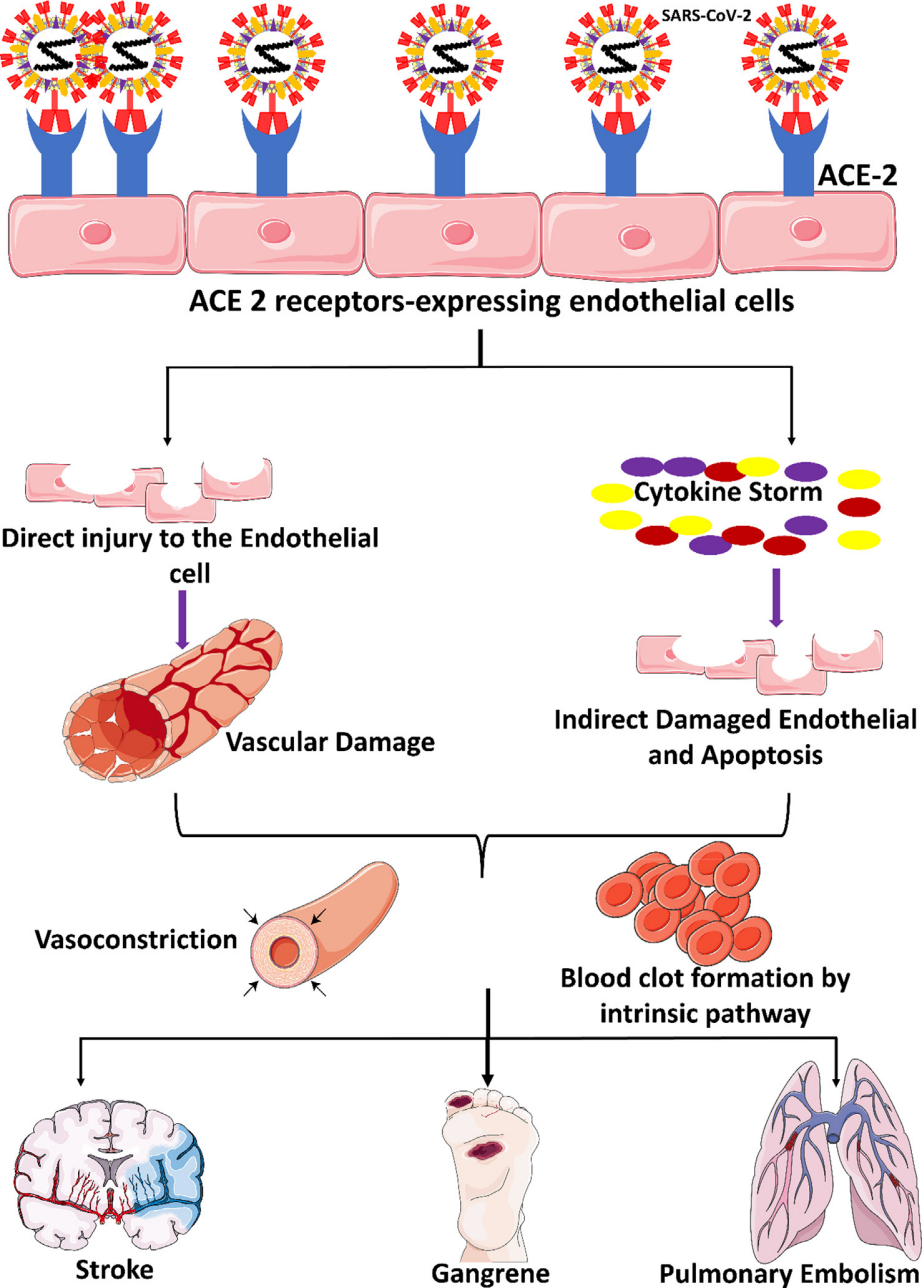
Demonstration of the mechanism of SARS-CoV-2 to cause gangrene and other vascular-related complications.

Seventy-eight years of COVID-19 positive older women had respiratory symptoms, brain fog, and discoloration at the extremities, particularly in hands, nose, and foot fingers. Reports confirmed from the laboratory displayed DIC. The discoloration of fingers, nose, and toes swiftly progressed into dry gangrene during the three days stay in hospital. Coma was followed after the deterioration of neurological state, and the patient died. The report also discussed that in serious subjects of COVID-19, the disease could get more intricated by ARDS, sepsis, and multi-organ dysfunction. This case showed that a non-vasculopathy patient develops dried gangrene due to COVID-19′s coagulopathy and disseminated intravascular coagulation (E et al., 2020). Literature also supports that SARS-COV-2 infection leads to hypercoagulability in different forms like gangrene, stroke, pulmonary embolism, and other acute thrombotic complications, thus approving the use of anticoagulant drugs. There are no guidelines for prophylactic and therapeutic anticoagulation timing and dosage in COVID-19 subjects. In COVID-19 patients, the susceptibility of catching thrombosis appears to be multifactorial, including proinflammatory condition, cytokine crisis, hypoxia-induced thrombus, cytopathological effects, and endothelium cell inflammation resulting in the development of intra alveolar or systemic fibrin clots (Singh et al., 2020). Hypothesis about how blood clot formation (which can further advance into thrombosis and gangrene) takes place in the COVID-19 patients’ states that “Due to an internal injury in the endothelium of blood vessels either directly by SARS-CoV-2 infection or by the virus-mediated inflammatory immune response, may result in vasoconstriction and the activation of coagulation and blood clotting pathways, resulting in the formation of blood clots”. (Biswas et al., 2021), This hypothesis is further demonstrated in Fig. 4. As of now, very few cases have been seen where gangrene is associated with COVID-19. This symptom is considered one of the rarest and needs more research to reach any specific conclusion.

## 8 Impact of COVID-19 on endothelial dysfunctioning

The endothelium is among the biggest organs in the entire human body (JP et al., 2000). Endothelial cellular damage participates in the pathology of multiple-organ collapse in COVID-19 leads to high BP (Pons et al., 2020) and nephrological disorders (Durvasula et al., 2020) mediated by the interaction with ACE-2 receptors present on the endothelial system. The protection of the CVS is mediated by endothelial cells (ECs), releasing the proteins that influence the blood clotting and immune system. Damage to the ECs results in extensive cardiovascular tissues damage, eventually causing spontaneous heart attacks in COVID-19. Moreover, injury to the ECs leads to inflammation in the blood vessels, causing plaque rupture and heart attack, and subsequent cytokine storm to inflammation-induced heart failure. The major contributing factors towards endothelial damage includes disbalance between antioxidants and production of ROS and RNS, left ventricle remodeling, fibrosis by releasing transforming growth factor-beta (TGFβ) by differentiated monocytes (Young et al., 2020).

## 9 Conclusion

COVID-19 has afflicted over 224 nations and territories, leading to widespread fatalities. It was observed that people with underlying chronic illnesses are more likely to get SARS-CoV-2 infections. Individuals with COVID-19 who have a past medical history of cardiovascular disorder, cancer, obesity, chronic lung disease, diabetes, or neurological disease had the worst prognosis and are more likely to develop ARDS or pneumonia. Furthermore, older people, patients with chronic kidney illness, and cancer are not only in danger of COVID-19 infection, but they also have a considerably higher mortality rate. COVID-19 indications vary from moderate respiratory sickness to serious illness requiring intubation and oxygen therapy. Because the patient may be asymptomatic and the incubation period is between 2 and 14 days, it is difficult to make a timely diagnosis. Unfortunately, if respiratory problems occur during this time, it is critical to seek medical attention immediately. Individuals with comorbidities should take all necessary steps to avoid SARS-CoV-2 infection and associated mortality. Regular handwashing with soap or using alcohol-based hand sanitizer, minimizing inperson contact and practicing social distance, wearing a face mask in public places, and avoiding going to public places unless essential are among the precautions. Individuals with comorbidities should be immunized as soon as possible, to prevent futher complications. Global public health effort is required to increase awareness about minimizing the burden of these comorbidities that cause fatalities in COVID-19 patients.

## Authors Contribution

**Jonaid Ahmad Malik:** Writing. **Sakeel Ahmed:** Writing. **Mrunal Shinde:** Writing. **Mohammad Hajaj Said Almermesh:** Editing and Review. **Saleh Alghamdi:** Conceptualization. **Arshad Hussain:** Supervision. **Sirajudheen Anwar:** Conceptualization, Editing and Supervision.

## Declaration of Competing Interest

The authors declare that they have no known competing financial interests or personal relationships that could have appeared to influence the work reported in this paper.
